# Complete Genome Sequences of *Mycobacterium kansasii* Strains Isolated from Rhesus Macaques

**DOI:** 10.1128/genomeA.00187-17

**Published:** 2017-04-20

**Authors:** Aruna Panda, Sushma Nagaraj, Xuechu Zhao, Hervé Tettelin, Louis J. DeTolla

**Affiliations:** a Department of Pathology, and Department of Epidemiology and Public Health, Program of Comparative Medicine, University of Maryland School of Medicine, Baltimore, Maryland, USA; b Department of Microbiology and Immunology, Institute for Genome Sciences, University of Maryland School of Medicine, Baltimore, Maryland, USA; c Department of Medicine, Division of Infectious Diseases, University of Maryland School of Medicine, Baltimore, Maryland, USA

## Abstract

*Mycobacterium kansasii* is a nontuberculous mycobacterium. It causes opportunistic infections with pulmonary and extrapulmonary manifestations. We report here the complete genome sequences of two *M. kansasii* strains isolated from rhesus macaques. We performed genome comparisons with human and environmental isolates of *M. kansasii* to assess the genomic diversity of this species.

## GENOME ANNOUNCEMENT

*Mycobacterium kansasii*, a nontuberculous mycobacterium, is an opportunistic pathogen of humans. It induces pulmonary or disseminated infections in humans infected with HIV. It is known to cause fibrocavitary lung disease in non-HIV patients (1–6). *M. kansasii* isolates have been recovered from environmental samples such as dust, soil, and water (7). The presence of *M. kansasii* has been infrequently reported from asymptomatic wild or domestic animals such as birds, wild deer, pigs, and dogs (8). Mycobacteriosis due to *M. kansasii* infection has been reported in monkeys (9, 10). Infection with *M. kansasii*, accompanied by inflamed lymph nodes or pneumonic lesions, has been described in rhesus monkeys, squirrel monkeys, cattle, llamas, goats, camels, and both domestic and feral pigs (9, 10).

We report here the complete genome sequences of two *M. kansasii* strains, 11-3469 and 11-3813, isolated from Chinese rhesus macaques utilized in biomedical research. Genomic DNA from each *M. kansasii* isolate was sequenced using the Pacific Biosciences RS II platform (two SMRT cells per genome; 183,133 reads with an average length of 2,600 nucleotides [nt] were obtained for strain 11-3469, and 133,528 reads with an average length of 2,678 nt were obtained for 11-3813). Reads were assembled using HGAP Assembler version 2.0.1 (11), resulting in 21 contigs with a cumulative size of 6,801,699 bp for 11-3469, and 18 contigs with a cumulative size of 6,629,039 bp for 11-3813. Annotation was performed using the IGS Prokaryotic Annotation Engine (12). The genome sequence for 11-3469 had a G+C content of 66.11%, 49 tRNA genes, three rRNA operons, and 8,533 predicted open reading frames, while that for 11-3813 had a G+C content of 66.09%, 47 tRNA genes, three rRNA operons, and 9,491 predicted open reading frames.

To explore the genomic diversity that exists between *M. kansasii* strains obtained from monkeys and humans or from the environment, we downloaded annotated *M. kansasii* genome sequences available in GenBank as of 24 January 2017. These included finished genome sequences of the human isolate type strain Hauduroy ATCC 12478 (CP006835.1) and two more human isolates, strains 662 from bronchial lavage (CP009481.1) and 824 from sputum (CP009483.1); draft genome sequences of strains 732 from human sputum (JANZ00000000.1) and SMC1 from a human-associated habitat (JNDJ00000000.1); and six environmental isolates from Europe: 1010001454, 1010001458, 1010001468, 1010001493, 1010001495, and 1010001469 (13).

Multiple whole-genome sequence alignments were performed with Mugsy version 1r2.3.1 software within the CloVR Comparative pipeline (14). Core segments, including single nucleotide polymorphisms, were analyzed with Phylomark version 1.1 software (15) and a neighbor-joining phylogenetic tree built using the MEGA7 software (16). The tree revealed two major clades, one consisting of all but one of the environmental isolates plus strain 732, and the other one composed of 1010001495, all remaining human isolates, and our monkey isolates. Monkey isolate 11-3469 was slightly more closely related to the human isolates than isolate 11-3813. We conclude that disease-causing human and monkey isolates are more closely related to each other than to environmental isolates.

### Accession number(s).

This whole-genome shotgun project has been deposited in GenBank under the accession numbers MVBM00000000 and MVBN00000000 for *M. kansasii* strains 11-3813 and 11-3469, respectively.
